# Genome of *Lamprobacter modestohalophilus* ShN*Lb*02, a moderate halophilic photosynthetic purple bacterium of the Chromatiaceae family

**DOI:** 10.1128/mra.00128-24

**Published:** 2024-03-25

**Authors:** John A. Kyndt, Irina A. Bryantseva, Vladimir M. Gorlenko, Johannes F. Imhoff

**Affiliations:** 1 College of Science and Technology, Bellevue University, Bellevue, Nebraska, USA; 2 Winogradsky Institute of Microbiology, Research Center of Biotechnology, Russian Academy of Sciences, Moscow, Russia; 3 GEOMAR Helmholtz Centre for Ocean Research Kiel, Kiel, Germany; California State University San Marcos, San Marcos, California, USA

**Keywords:** *Lamprobacter*, Lake Shunet, *Halochormatium*, whole-genome sequence

## Abstract

The genome sequence of the moderately halophilic *Lamprobacter modestohalophilus* ShN*Lb*02 was compared to those of other *Lamprobacter* and *Halochromatium* species. It revealed an average nucleotide identity of 94% to *Lpb. modestohalophilus* DSM 25653 and of 89.7% to *Halochromatium roseum* DSM 18859, underscoring their close phylogenetic relationship.

## ANNOUNCEMENT

The genus *Lamprobacter* contains a single species of halophilic purple sulfur bacteria. Previous morphological, physiological, and 16S rRNA studies have shown a close relationship of *Lamprobacter modestohalophilus* to *Halochromatium roseum* and more distantly to *Halochromatium salexigens* and *Halochromatium glycolicum*. These studies have led to the proposal to move *Hch. roseum* to the genus *Lamprobacter* (1). Strain ShN*Lb*02 is the only *Lamprobacter* strain that does not require vitamin B_12_ for growth (1). The genome sequence of *Lpb. modestohalophilus* DSM 25653 had previously been determined (2). We now report the sequence of strain ShN*Lb*02, which will allow a refined phylogenetic comparison of the genus.

Strain ShN*Lb*02 was isolated from the meromictic saline Lake Shunet (Russia, Siberia; 54°25′07″N; 90°13′41″E; salinity 65 g/L) by serial dilutions after repeated transfers of well-isolated colonies from 0.5%–0.7% (wt/vol) agar medium as described in references (1, 3). Genomic DNA was prepared from frozen cells stored in anaerobic vials at −80°C, using the GeneJET DNA purification kit (Thermo Scientific), giving an *A*_260/280_ of 1.81. The sequencing library was prepared using the Illumina DNA Library Prep kit. The genome was sequenced by an Illumina MiniSeq using 500 µL of a 1.8 pM library. Paired-end (2 × 150 bp) sequencing generated 2,472,406 reads and 193 Mbps. Quality control of the reads was performed using FASTQC (v1.0.0), using a k-mer size of 5 and contamination filtering for overrepresented sequences against the default contamination list. Oxford Nanopore library prep was performed following the Ligation Sequencing Kit (SQK-LSK110) without size selection, on a FLO-MIN106D flow cell with a MinION-Mk1B instrument (4). No DNA shearing was performed. Read QC and “superaccuracy basecalling” were performed using Guppy (v6.5.7) (5). We obtained 52,757 reads (74.4 Mbps), with a mean read length of 1,883 bp. A combined *de novo* genome assembly with the Illumina sequencing was performed using Unicycler (v0.5.0) (6) within BV-BRC (7). The resulting contig, CDS, and N50 values are in (Table 1). The coarse and fine consistency were 94% and 90%, respectively (8). The final assembled genome was 100% complete according to CheckM (v1.1.6) (9) with less than 2% contamination. The genome was annotated by the NCBI PGAP (v6.6) (10). Default parameters were used unless otherwise specified.

**TABLE 1:** Overview of genome features of all of the sequenced *Lamprobacter* and *Halochromatium* species^*a*^

Species	Size	% GC	Contigs	Coverage	N50	CDS	tRNAs	ANI	Reference	Genbank acc. #
*Lamprobacter modestohalophilus* ShN*Lb*02	6.1 Mb	61.8	606	44×	80,713	5,935	43	–	This study	JAXUFI010000000
*Lamprobacter modestohalophilus* DSM 25653	5.7 Mb	62	209	126×	90,783	5,899	47	94.4	(2)	NRRY00000000
*Halochromatium roseum* DSM 18859 [T]	5.2 Mb	61.9	88	50×	1,43,744	5,208	44	89.7	(2)	NHSH00000000
*Lamprobacter* sp. SM2E	6.2 Mb	61.4	787	55×	34,435	6,915	56	84	(11)	JAYESN000000000
*Halochromatium salexigens* DSM 4395 [T]	3.8 Mb	63.7	100	61×	66,939	3,831	46	81.5	(2)	NHSF00000000
*Halochromatium glycolicum* DSM 11080 [T]	5.3 Mb	64.6	147	75×	84,551	5,291	45	78.1	(2)	NRSJ00000000

^*a*^ ANI percentage is based on bidirectional ANIb values to strain ShN*Lb*02, calculated using JSpecies.

**Fig 1:**
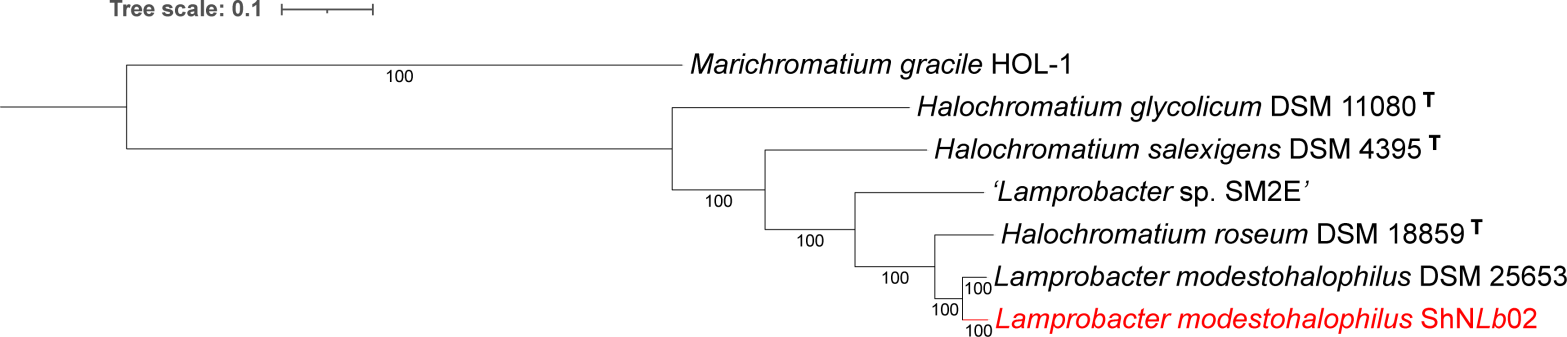
Phylogenetic tree of *Lamprobacter* whole-genome comparison to its closest relatives. The phylogenetic tree was generated using the codon tree method within BV-BRC (7), which used PGFams as homology groups and analyzed 500 aligned proteins and coding DNA from single-copy genes using RAxML (**V8**) (13, 14). The support values for the phylogenetic tree are generated using 100 rounds of the “Rapid bootstrapping” option of RaxML. *Marichromatium gracile* HOL-1 (17) was used as an outgroup. iTOL was used for the tree visualization (18).

A JSpecies comparison [12; v4.1.1] of average percentage nucleotide identity (ANI) gave 94.4% identity of *Lpb. modestohalophilus* ShN*Lb*02 to *Lpb. modestohalophilus* DSM 25653 and 89.7% identity to *Hch. roseum* DSM 18859. Phylogenetic analysis of the *Lpb. modestohalophilus* ShN*Lb*02 genome using RAxML (13, 14) showed *Lpb. modestohalophilus* DSM 25653 as the closest relative (Fig. 1) and more distantly *Hch. roseum* DSM18859 from saltpans in India (11) and “*Lamprobacter* sp. SM2E” from the Nebraska Salt Marshes (15). Both the ANI and phylogenetic comparisons support the earlier proposition, based on morphological and physiological data, of including *Hch. roseum* DSM 18859 into the *Lamprobacter* genus (1). All genomes from *Lpb. modestohalophilus* and *Hch. roseum* contain a complete gas vesicle operon, which is absent from *Hch. salexigens*. The ShN*Lb*02 genome contains three distinct gene clusters for cobalamin biosynthesis, with an AAI of 64% using LALIGN (16), consistent with the observation that ShN*Lb*02 does not require vitamin B_12_ supplement for growth (1).

## Data Availability

This Whole-Genome Shotgun project has been deposited at DDBJ/ENA/GenBank under the accession JAXUFI000000000. The version described in this paper is version JAXUFI010000000. The raw sequencing reads have been submitted to SRA, and the corresponding accession number for the Illumina data is SRR27256383. The accession numbers for the Oxford Nanopore data are SRR27887957, SRR27887954, SRR27887951, SRR27887959, SRR27887956, SRR27887953, SRR27887950, SRR27887958, SRR27887955, SRR27887952, SRR27887949, SRR27887948, SRR27887947, SRR27887946.
